# Termination of pregnancy after a prenatal diagnosis of congenital diaphragmatic hernia: Factors influencing the parental decision process

**DOI:** 10.1002/pd.6274

**Published:** 2022-12-04

**Authors:** Emily J. J. Horn‐Oudshoorn, Nina C. J. Peters, Arie Franx, Alex J. Eggink, Suzan C. M. Cochius‐den Otter, Irwin K. M. Reiss, Philip L. J. DeKoninck

**Affiliations:** ^1^ Department of Paediatrics Division of Neonatology Erasmus MC University Medical Centre Rotterdam The Netherlands; ^2^ Department of Obstetrics and Gynaecology Division of Obstetrics and Fetal Medicine Erasmus MC University Medical Centre—Sophia Children's Hospital Rotterdam The Netherlands; ^3^ Intensive Care and Department of Paediatric Surgery Erasmus MC University Medical Centre Rotterdam The Netherlands

## Abstract

**Objective:**

To evaluate the incidence of termination of pregnancies (TOP) and factors associated with the decision for TOP in prenatally detected congenital diaphragmatic hernia (CDH).

**Study Design:**

Single‐centre retrospective cohort includes all prenatally detected CDH cases born between January 2009 and December 2021. Parental factors, such as parity, and fetal characteristics, such as disease severity, were collected. Descriptive statistics were used to present the data. Differences between terminated and continued pregnancies were analysed.

**Results:**

The study population consisted of 278 prenatally detected CDH cases of which 80% detected <24 weeks of gestation. The TOP rate was 28% in cases that were detected <24 weeks of gestation. Twenty continued pregnancies resulted in either intrauterine fetal demise (*n* = 6), preterm birth <24 weeks (*n* = 2), or comfort care after birth (*n* = 12). The survival rate was 70% in the remaining 195 live born cases. Factors associated with the decision for TOP were additional fetal genetic or anatomical abnormalities (*p* < 0.0001) and expected severity of pulmonary hypoplasia in left‐sided CDH (*p* = 0.0456).

**Conclusion:**

The decision to terminate a pregnancy complicated by fetal CDH depends on the severity of pulmonary hypoplasia and the presence of additional abnormalities. This emphasises the importance of early referral to expertise centres for detailed evaluation and multidisciplinary counselling.

## Key points

What is already known about this topic? What does this study add?

- Implementation of routine structural ultrasound evaluations has increased prenatal detection of congenital birth defects, providing parents the option to reflect about termination of pregnancy (TOP).
- The incidence of TOP in case of fetal congenital diaphragmatic hernia varies between 6% and 100% in historical cohorts; in our cohort spanning over a decade, this was 28%.
- The parental decision on discontinuing the pregnancy was associated with a higher estimated disease severity, but not with parent‐specific factors.

## 1 INTRODUCTION

The implementation of routine prenatal ultrasound screening programs in combination with technological improvements in scanning equipment has led to an increase in the prenatal detection rate of congenital birth defects.^1^ Currently, around 70% of all cases with a congenital diaphragmatic hernia (CDH) are detected before birth of which 3%–29% in the first trimester, 58%–72% in the second trimester, and 8%–25% in the third trimester.^1^, ^2^, ^3^ Early detection of congenital malformations provides parents an opportunity to reflect on whether to continue the pregnancy or not. Legislation in the Netherlands obligates that a decision to end the pregnancy must be made prior to 24 weeks of gestation with an exception for conditions that have an unquestionable lethality after birth. However, late termination of pregnancy (TOP) in the latter group is rarely done because of stringent administrative procedures and post‐hoc ethical reviews.

The reported TOP rates for CDH cases vary between historical cohorts from different countries with observations ranging between 6% and 100%.^2^, ^4^, ^5^, ^6^, ^7^, ^8^, ^9^, ^10^, ^11^, ^12^, ^13^ This large variation is partly explained by differences in study populations as the TOP rate is lower in populations that include cases in which TOP was no longer an option due to a late diagnosis.

Specific fetal characteristics are associated with adverse outcomes for infants with CDH, such as gestational age at diagnosis,^1^ additional genetic or anatomical abnormalities,^14^, ^15^, ^16^ a right‐sided defect,^15^, ^17^, ^18^, ^19^, ^20^ expected severe lung hypoplasia (determined with ultrasound or fetal magnetic resonance imaging),^21^ and intrathoracic liver position.^18^, ^21^, ^22^, ^23^, ^24^ These disease‐related factors might play a role for families when deciding to either continue or terminate the pregnancy, as was observed in other fetal abnormalities.^25^, ^26^, ^27^, ^28^, ^29^ Parental characteristics on the other hand, such as maternal age, parity, ethnicity, and socioeconomic factors, might also play a role.^26^, ^27^, ^30^, ^31^, ^32^, ^33^ In this single‐centre retrospective study, we describe a cohort of prenatally detected CDH patients and evaluate fetal and parental factors that may be associated with a decision to discontinue the pregnancy.

## 2 METHODS

This is a single‐centre retrospective cohort study performed at Erasmus MC, University Medical Centre Rotterdam, The Netherlands, a national and level 3 referral centre. Data were retrospectively extracted from the medical files of consecutive cases evaluated at our centre between January 2009 and December 2021. The study population consisted of all cases with a prenatal diagnosis of CDH that received prenatal counselling and for whom birth and postnatal management were planned at our centre. The research protocol was approved by the medical ethical committee (METC 2022‐0340) of the Erasmus MC and informed consent was waived due to the retrospective study design.

The following maternal data were collected: age at delivery, mode of conception (spontaneous or assisted), parity, ethnic background (Caucasian or other), socioeconomic status (low, middle, or high), and relationship status. Socioeconomic status was defined by maternal educational level: low (no education, primary school, and lower vocational training), middle (intermediate vocational training), or high (higher vocational training or university). Fetal characteristics concerned gestational age at diagnosis or at first visit to our centre, side of the hernia (left or right), observed to expected lung‐to‐head ratio (o/e LHR) and gestational age at measurement, liver position (intra‐abdominal or intrathoracic), additional anatomical and genetic abnormalities detected before 24 weeks of gestation, fetal surgery, and gestational age at birth. Fetal surgery in the form of tracheal occlusion was offered to parents throughout the entire study period. Neonatal outcomes included overall mortality and neonatal death (death within the first 28 days after birth). If possible, we collected the o/e LHR measurement that was used for the prenatal counselling. Left‐sided CDH cases were divided in expected *mild* (o/e LHR 35.0%–44.9% with intra‐abdominal liver, or o/e LHR ≥45.0%, irrespective of liver position), *moderate* (o/e LHR 25.0%–34.9%, irrespective of liver position, or o/e LHR 35.0%–44.9% with intrathoracic liver), or *severe* (o/e LHR <25.0%, irrespective of liver position) pulmonary hypoplasia.^21^, ^34^ Right‐sided CDH cases were divided in expected *moderate* (o/e LHR ≥50.0%) or *severe* (o/e LHR <50.0) pulmonary hypoplasia.^17^, ^20^

Normality of the data was checked with quantile‐quantile plots and density distributions combined with the Shapiro‐Wilk test. Continuous data were presented as mean ± standard deviation or median [interquartile range], depending on whether the data were normally distributed. Categorical data were presented as absolute numbers and percentages. Statistical tests for continuous data were the Mann‐Whitney *U* test (non‐parametric) and the Student *t* test (parametric). Categorical variables were analysed with a chi‐squared test or Fisher exact test. Statistical analysis was done using R (R Core Team, 2020). *p* < 0.05 was considered statistically significant.

## 3 RESULTS

**FIGURE 1:**
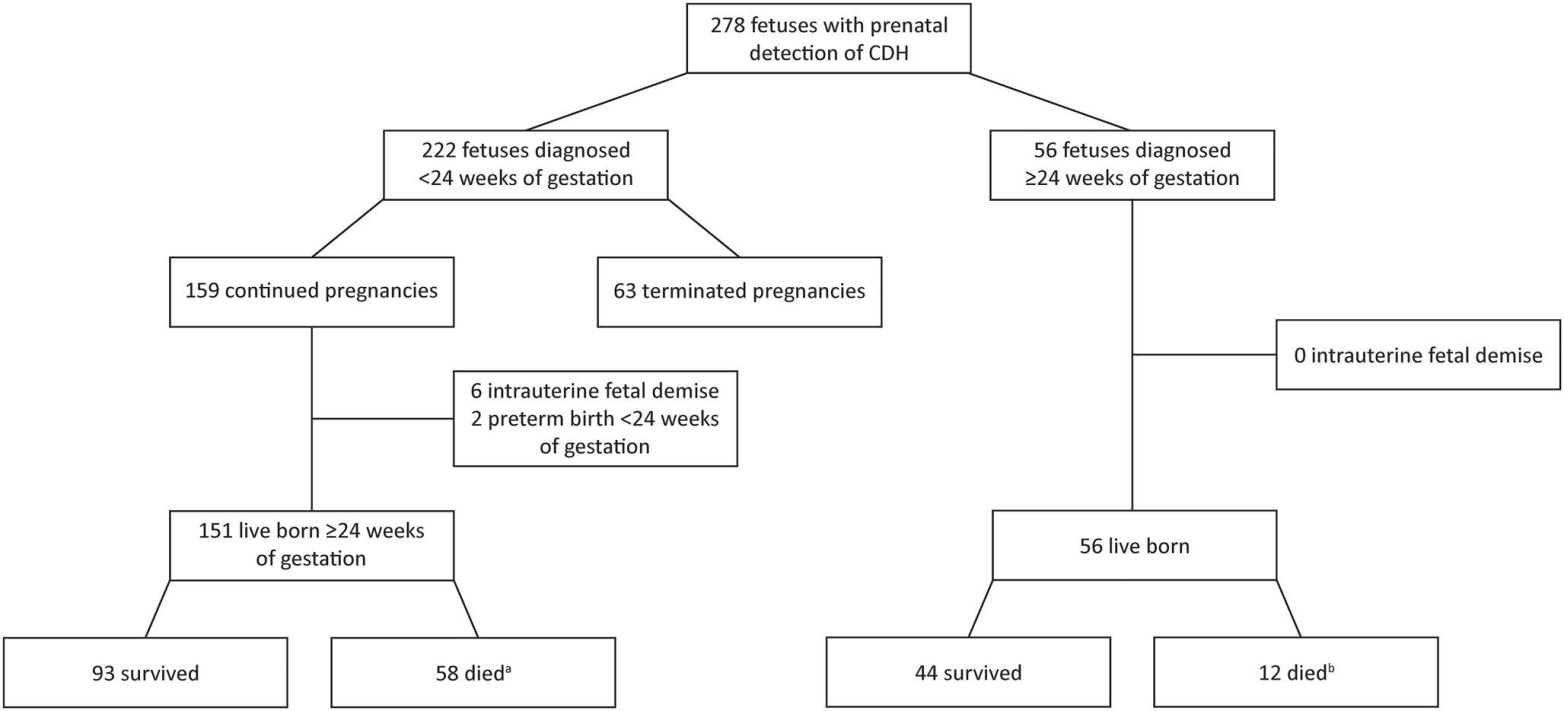
Flowchart of study population. CGH, congenital diaphragmatic hernia. ^a^9 infants received comfort care after birth. ^b^3 infants received comfort care after birth.

A total of 278 pregnancies with a prenatal diagnosis of a CDH were included (Figure 1). Eighty percent (*n* = 222) were detected before 24 weeks of gestation (*early* diagnosis). In *early* diagnosed cases, the expected severity of pulmonary hypoplasia was mild in 41% (*n* = 90), moderate in 34% (*n* = 76), and severe in 18% (*n* = 41). In a small proportion of cases (7%, *n* = 15), the o/e LHR could not be determined because of early gestational age. In cases diagnosed ≥24 weeks of gestation (*late* diagnosis), the expected severity of pulmonary hypoplasia was mild in 55% (*n* = 31), moderate in 27% (*n* = 15), and severe in 18% (*n* = 10). Prenatal treatment in the form of fetoscopic endoluminal tracheal occlusion (FETO) was attempted in 11 early diagnosed cases, but in one case, it was not possible to position the tracheal balloon correctly.

**FIGURE 2:**
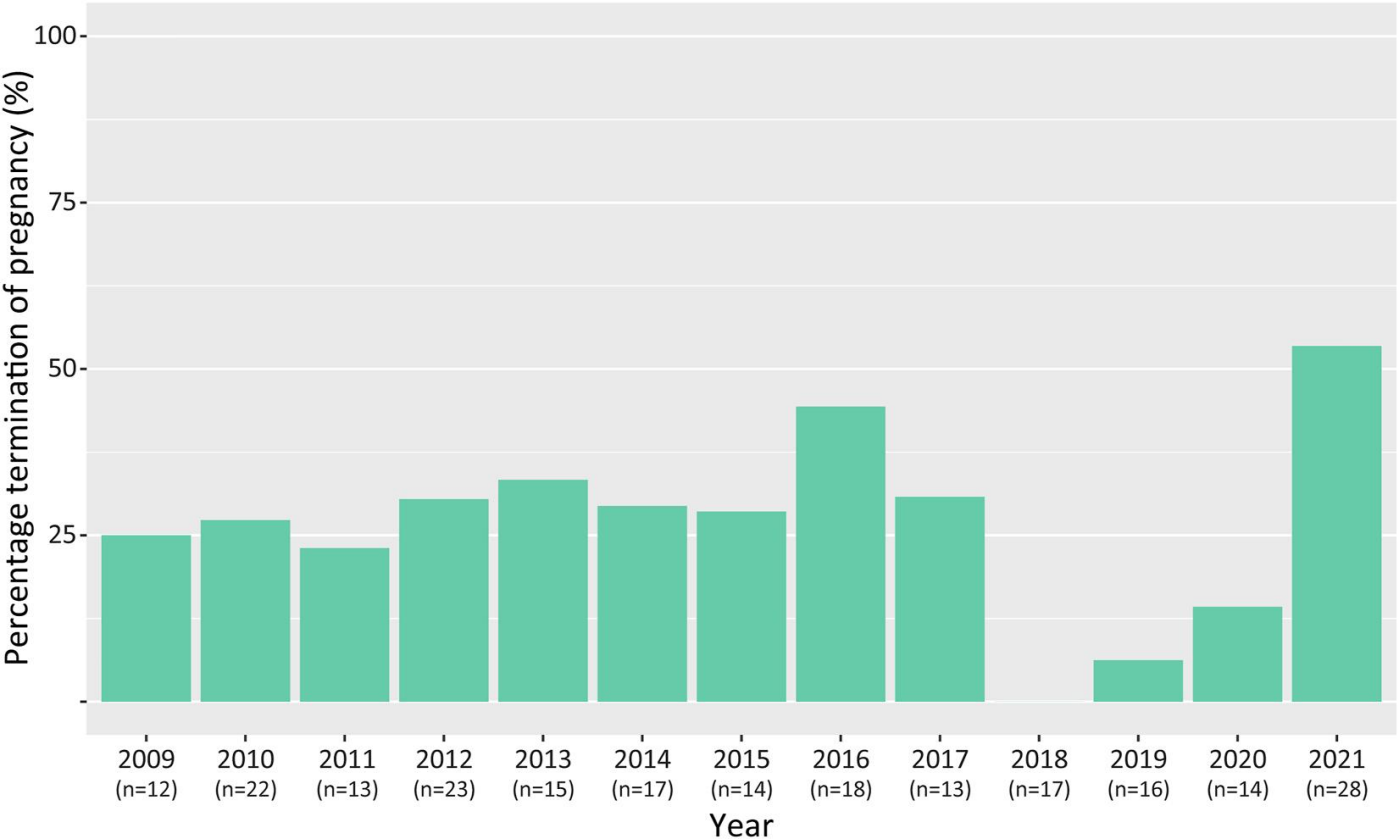
Termination of pregnancy rates in early (<24 weeks of gestation) detected congenital diaphragmatic hernia. Number of early detected congenital diaphragmatic cases per year are depicted between brackets.

The TOP rate was 28% (63/222) in all *early* diagnosed cases and 23% (63/278) in the entire study population. Figure 2 demonstrates the TOP rate per year in all *early* diagnosed cases, ranging between 0% and 54%. Of 215 continued pregnancies, 6 pregnancies resulted in intrauterine fetal demise, 2 pregnancies resulted in preterm birth prior to 24 weeks of gestation, and in 12 pregnancies, the infants received comfort care after birth. The majority (*n* = 10) of these infants receiving comfort care were diagnosed with additional abnormalities before 24 weeks of gestation, but parents refrained from TOP. Additionally, 1 infant with isolated CDH was born very preterm (24^+5^ weeks of gestation) and received comfort care because of that. The remaining 195 infants were born at a median gestational age of 38^+1^ [37^+1^‐38^+4^] weeks, reflecting the standard‐of‐care in our centre with induction of labour around 38 weeks of gestation. The overall survival rate in live born cases receiving active management after birth was 70% (137/195) with non‐surviving infants dying on a median of day 12 [2–33]; the majority (67%) already died within the first 28 days after birth. The expected severity of pulmonary hypoplasia in non‐surviving infants was mild in 21% (*n* = 12), moderate in 43% (*n* = 25), and severe in 36% (*n* = 21).

Table 1 depicts the characteristics for both terminated pregnancies and continued pregnancies in *early* diagnosed cases. Despite numerical differences, no significant differences were observed in parental factors. The odds of pregnancy termination were higher for cases with at least one additional abnormality (OR 7.78; 95% CI [4.03–15.00]; *p* < 0.0001). In left‐sided CDH, o/e LHR was significantly lower in terminated pregnancies (35% [29%–40%] vs. 42% [33%–50%], *p* = 0.00372), but liver position was not significantly different between terminated and continued pregnancies (intrathoracic liver position in 46% vs. 49%, *p* = 0.895). The expected severity of pulmonary hypoplasia in left‐sided CDH, combining liver position and o/e LHR, was significantly different between the two groups (*p* = 0.0456) with a higher percentage of moderate (50% vs. 36%) and severe (16% vs. 9%) pulmonary hypoplasia in the group with terminated pregnancies. In right‐sided CDH, o/e LHR was not significantly different (48% [42%–50%] vs. 35% [28%–49%], *p* = 0.159). In the total study population, the TOP rates per severity group were 17% for mild CDH, 30% for moderate CDH, and 34% for severe CDH.

**TABLE 1:** Characteristics of terminated and continued pregnancies in early (<24 weeks of gestation) detected congenital diaphragmatic hernia

	Terminated pregnancies		Continued pregnancies		*p*‐value
	(*n* = 63)		(*n* = 159)		
Maternal age at delivery (years)		32 ± 5		31 ± 6	0.487
Nulliparous women		29 (46)		83 (52)	0.497
Spontaneous conception	*n* = 61	52 (85)	*n* = 158	146 (92)	0.175
Caucasian	*n* = 58	43 (74)	*n* = 158	103 (65)	0.280
Socioeconomic status	*n* = 35		*n* = 131		0.664
Low		10 (29)		48 (37)	
Middle		14 (40)		45 (34)	
High		11 (31)		38 (29)	
Single mothers		2 (3)		4 (3)	1
Gestational age at diagnosis diaphragmatic defect (weeks)		20^+1^ [19^+1^–21^+1^]		20^+4^ [20^+0^–21^+1^]	0.061
Left‐sided defect	*n* = 61	52 (85)	*n* = 157	138 (88)	0.764
Additional abnormalities		38 (60)		26 (16)	<0.0001
Anatomic		33 (52)		20 (13)	
Genetic		22 (35)		14 (9)	
Both anatomic and genetic		16 (25)		6 (4)	

*Note*: Data are expressed as mean ± standard deviation, median [interquartile range], or *N* (%).

## 4 DISCUSSION

To the best of our knowledge, this is the first series that evaluated factors associated with the parental decision to discontinue the pregnancy in case of a CDH. In this study, we observed that around one fourth of parents decided not to continue the pregnancy. Factors associated with the decision of terminating the pregnancy were the expected severity of pulmonary hypoplasia in left‐sided CDH and the presence of additional abnormalities.

Previous series have observed that the presence of additional anatomical and/or genetic abnormalities influences the parental decision on TOP in fetuses with CDH with TOP rates of 6%–19% for isolated CDH cases and 35%–61% for non‐isolated cases.^7^, ^13^, ^35^ The findings in our cohort reconfirm this, observing a threefold increase in TOP (16% vs. 59%) in non‐isolated CDH cases, highlighting the importance of prenatal genetic testing and expert ultrasound examination. On the other hand, genetic testing is rapidly progressing to whole exome sequencing or even whole genome sequencing for any congenital abnormality. This in turn will increase the yield in detecting potential genetic aberrations of unknown significance; yet the challenge is to determine the clinical relevance of each of these incidental findings.^36^, ^37^ The clinical implications of these variants of unknown significance should be further assessed using international databases, aiming at aiding parents in making decisions about the current but also future pregnancies.^38^

As expected based on historical literature in other fetal conditions, disease severity seems to be a factor in parental decision‐making.^25^, ^26^, ^28^, ^33^ In CDH, disease severity is estimated based on the combination of side of the defect, position of the liver, and estimated lung size. Although we did not find significant differences in the first two respective factors, the combination of side of the defect, position of the liver, and estimated lung size was significantly different in left‐sided CDH with a lower incidence of mild and higher incidences of moderate and severe pulmonary hypoplasia in terminated pregnancies.

The option of fetal therapy was discussed with families during the whole study period; yet until 2020, this was only offered within a randomised trial and participation required temporary relocation to a FETO centre abroad. It is unfortunately impossible to determine whether these considerations may have influenced the decision process. With the recently proven benefits of fetal therapy for isolated cases with severe pulmonary hypoplasia and minimal maternal risks, we speculate that more parents will opt for fetal therapy, which may affect the TOP rate in this subgroup.^39^ On the other hand, despite improved survival rates, infants with severe CDH will still need a lengthy period of intensive care and complications are not uncommon. The psychological and social impact of this prolonged period of uncertainty and anxiety on the whole family should not be underestimated, and for some parents, this will be the main consideration to discontinue the pregnancy.

Clinically relevant differences have been demonstrated between disease severity assessment in referring hospitals and expertise referral centres.^10^ In case of a prenatal diagnosis of a CDH, prompt referral to a specialised centre with an experience in both prenatal and postnatal care of CDH would therefore provide parents with the much needed comprehensive and reliable information.^40^ Thus, healthcare professionals should be cautious when counselling parents without having consulted a healthcare professional with expertise on CDH.^10^, ^41^ Moreover, to ensure parental wellbeing, psychosocial support should be integrated in the prenatal trajectory.^40^

Routinely, a structural ultrasonographic examination is offered to all pregnant women in the Netherlands around 20 weeks of gestation. However, a 13‐week ultrasound has recently (2021) become available as part of an implementation study (Implementation of first Trimester Anomaly Scan [IMITAS‐study]), potentially resulting in an earlier gestational age at diagnosis. As there is evidence that early TOP is less traumatic for the parents, this is certainly interesting for women expecting a child with CDH.^42^ On the other hand, earlier studies have observed relatively low detection rates of CDH in the first trimester; hence, the effect of implementing a routine 13‐weeks screening ultrasound on the incidence of TOP is uncertain.^3^

In contrast to previous series, we could not confirm parental factors that were significantly associated with TOP.^27^ Despite an apparent higher parity of mothers in the TOP‐group, this difference did not reach significance, underlining conflicting results in earlier studies.^31^

In our cohort, the survival rate was 49% (137/278) in the entire study population, 64% (137/215) in all continued pregnancies, and 70% (137/195) in all live born cases receiving active postnatal management. These different survival rates reflect the hidden mortality when only the survival rate in live born cases is reported. It should be noted that our single‐centre results may not be representative of other centres or countries due to differences in regulations regarding pregnancy termination, cultural or religious influences, and prenatal counselling.^27^ We speculate that our cohort depicts an overall lesser severity of pulmonary hypoplasia in live born cases than other centres, due to the higher likelihood of severe cases to be terminated.^43^, ^44^ On the other hand, the improvement in survival of infants with CDH in the past decades might have resulted in a tendency for women to continue their pregnancy.^7^ However, in our single‐centre series, we could not determine any trend in TOP rates.

Interestingly, we observed lower TOP rates between 2018 and 2020. These lower TOP rates may be due to an increased number of intrauterine fetal demise, preterm birth prior to 24 weeks of gestation, and comfort care after birth in these specific years. Indeed, 4 of 6 cases resulting in intrauterine fetal demise, 1 of 2 cases resulting in preterm birth prior to 24 weeks of gestation, and 4 of 9 cases receiving comfort care after birth concerned pregnancies in 2018–2020. Another explanation might be the estimated severity of pulmonary hypoplasia. This could particularly be the case in 2019, as we did not have any case with expected *severe* pulmonary hypoplasia in that year.

Unfortunately, we were unable to retrieve data on the exact process of parental decision‐making, and thus, we should be cautious in drawing firm conclusions. In this study, we opted not to use parental questionnaires as we expected both recall bias and selection bias due to more responses from parents who continued their pregnancies or from parents who have only recently been confronted with a prenatal diagnosis of CDH. Another limitation of this retrospective study is that we were unable to adjust for the influence of counselling by specific healthcare professionals.^45^ However, parental counselling in our centre is routinely performed by both a maternal‐fetal medicine specialist and a paediatric specialist, and thus, we believe that this influence is limited. Although it is standard‐of‐care to refer parents with a fetal diagnosis of CDH to an expertise centre, we cannot rule out the possibility of other centres performing TOP in prenatally diagnosed CDH without referral. Thus, our estimated TOP rate might be a slight underestimation. As already mentioned, the single‐centre study design hampers translation of our results to other centres.

## 5 CONCLUSIONS

Parental decision‐making on whether to continue or terminate a pregnancy with a fetal diagnosis of CDH is a delicate process that is mainly influenced by fetal disease severity and the presence of additional fetal abnormalities. To guarantee counselling of parents with reliable information on disease severity, we advise that parental counselling should be carried out in dedicated referral centres with multidisciplinary expertise on these important prenatal factors.

## CONFLICT OF INTEREST

The authors have nothing to declare.

## ETHICS STATEMENT

The research protocol was approved by the local medical ethical committee (METC 2022‐0340) and informed consent was waived due to the retrospective study design.

## Data Availability

Data are available upon reasonable request to the corresponding author.
